# Adoption of Electronic Medical Records for Chronic Disease Care in Kenyan Refugee Camps: Quantitative and Qualitative Prospective Evaluation

**DOI:** 10.2196/43878

**Published:** 2023-10-05

**Authors:** Emily Lyles, Kenneth Paik, John Kiogora, Husna Hussein, Alejandra Cordero Morales, Lilian Kiapi, Shannon Doocy

**Affiliations:** 1 Department of International Health, Johns Hopkins Bloomberg School of Public Health, Baltimore, MD, United States; 2 Institute for Medical Engineering and Science, Massachusetts Institute of Technology, Cambridge, MA, United States; 3 International Rescue Committee, Nairobi, Kenya; 4 International Rescue Committee, London, United Kingdom

**Keywords:** mHealth, hypertension, diabetes, chronic disease, Kenya, refugees, mobile health, noncommunicable disease

## Abstract

**Background:**

Noncommunicable disease (NCD) prevention and control in humanitarian emergencies is a well-recognized need, but there is little evidence to guide responses, leading to varying care delivery. The Sana.NCD mobile health (mHealth) app, initially developed in Lebanon, is the only known mHealth tool for NCD management designed to increase care quality and coverage for providers in humanitarian settings.

**Objective:**

We evaluated a specialized mHealth app consisting of an abbreviated medical record for patients with hypertension or diabetes, adapted for a Kenyan refugee camp setting.

**Methods:**

We tested an adapted version of the Sana.NCD app (diabetes and hypertension medical record) in an 11-month (May 2021 to March 2022) quantitative and qualitative prospective evaluation in Kenya’s Hagadera refugee camp. Leveraging the rollout of a general electronic medical record (EMR) system in the Kakuma refugee camp, we compared a specialized NCD management app to a general EMR. We analyzed secondary data collected from the Sana.NCD app for 1539 patients, EMR data for 68 patients with NCD from Kakuma’s surgical and outpatient departments, and key informant interviews that focused on Hagadera clinic staff perceptions of the Sana.NCD app.

**Results:**

The Hagadera NCD clinic reported 18,801 consultations, 42.1% (n=7918) of which were reported in the NCD app. The Kakuma EMR reported 350,776 visits, of which 9385 (2.7%) were for NCDs (n=4264, 1.2% hypertension; n=2415, 0.7% diabetes). The completeness of reporting was used as a quality-of-care metric. Age, sex, prescribed medicines, random blood sugar, and smoking status were consistently reported in both the NCD app (>98%) and EMR (100%), whereas comorbidities, complications, hemoglobin A_1c_, and diet were rarely reported in either platform (≤7% NCD app; 0% EMR). The number of visits, BMI, physical activity, and next visit were frequently reported in the NCD app (≥99%) but not in the EMR (≤15%). In the NCD app, the completeness of reporting was high across the implementation period, with little meaningful change. Although not significantly changed during the study, elevated blood sugar (*P*=.82) and blood pressure (*P*=.12) were reported for sizable proportions of patients in the first (302/481, 62.8%, and 599/1094, 54.8%, respectively) and last (374/602, 62.1%, and 720/1395, 51.6%, respectively) study quarters. Providers were satisfied with the app, as it standardized patient information and made consultations easier. Providers also indicated that access to historic patient information was easier, benefiting NCD control and follow-up.

**Conclusions:**

A specialized record for NCDs outperformed a more general record intended for use in all patients in terms of reporting completeness. This CommCare-based NCD app can easily be rolled out in similar humanitarian settings with minimal adaptation. However, the adaptation of technologies to the local context and use case is critical for uptake and ensuring that workflows and time burden do not outweigh the benefits of EMRs.

## Introduction

Access to and quality of health services are major concerns in conflict settings, where the destruction of health facilities, inadequate human resources for health, and displacement limit access to and quality of care. Innovative approaches to support facility and community care using digital solutions may help address these challenges and improve disease management in low-resource settings [1]. This is particularly important for noncommunicable diseases (NCDs), which require long-term care and are often complicated to manage. An estimated 41 million deaths due to NCDs occur annually, accounting for 71% of global mortality. Mortality disproportionately affects the poorest and most disenfranchised populations, with 85% of premature deaths due to NCDs among adults aged 30-69 years occurring in low- and middle-income countries [2].

Historically, humanitarian health interventions have not focused on NCDs; however, with demographic and epidemiologic transitions and increasing NCD burden, it is essential to address this gap. The need for NCD prevention and control in humanitarian emergencies is well recognized, but there is little evidence to guide the development of operational strategies or policies, resulting in varying delivery of NCD care [3,4]. Mobile health (mHealth) interventions are increasingly common and, with the widespread availability of devices, have the potential to overcome infrastructure, human resource, and access limitations in conflict settings [3,5]. Evidence for mHealth interventions, and electronic medical records (EMRs) specifically, is strong in high-income countries, where the need for context-specific modification of existing EMR models and the continued adaptability of these models to meet evolving clinical needs and environments is well established [6,7]. Although EMRs are increasingly common, little is empirically known about patient outcomes within these systems and the overall evidence base for EMRs, and mHealth interventions are generally less developed in low- and middle-income settings, particularly for decision support functions [8-10]. Decision support algorithms provide guidance to less-trained providers who are compelled to deliver care in the absence of physicians, increasing access to services and improving the quality of delivery. Enhanced recordkeeping is an important first step to improve the quality of care, and persistent digital health information can also improve the continuity of care over time and place in conflict and displacement contexts.

To date, the use of mHealth tools for NCDs in humanitarian settings has been limited and concentrated in the Middle East, specifically in Lebanon and Jordan. A 2012 pilot of cohort monitoring among Palestinian refugees with diabetes and hypertension at an UNRWA (United Nations Relief and Works Agency for Palestine Refugees in the Near East) primary health care clinic in Jordan showed that EMRs were a valuable monitoring mechanism, with potential to improve the quality and continuity of care for diabetes and hypertension [11,12]. The expansion of UNRWA’s EMR system to additional health centers further demonstrated the system’s ability to accurately capture service provision data and health outcomes [13,14]. In Iraq, a mobile diabetes self-management system that incorporates mobile glucose self-monitoring and a remote web interface and health management system was also found to lower hemoglobin A_1c_ (HbA_1c_) in a case study of 12 patients with type 2 diabetes [15].

More recently, a randomized controlled trial was conducted in rural areas and refugee camps in Lebanon, assessing the effectiveness of an integrative NCD intervention that includes weekly SMS text messages containing medical information and appointment or follow-up reminders [16]. The study observed improved health outcomes, namely blood pressure and HbA_1c_, among patients who received weekly SMS text messages for 1 year and concluded that the intervention was a simple, socially acceptable, and low-cost mechanism for improving NCD care. The SMS messaging intervention did not show as much promise in effecting behavior change, with fewer than one-third of participants reporting improvement in behaviors for managing diabetes or hypertension [17].

The Sana.NCD mHealth app (previously titled Sana.PCHR), developed by the Massachusetts Institute of Technology (MIT) Sana Mobile group, was initially designed for Syrian refugees in Lebanon. It is the only known mHealth tool for NCD management designed to increase the coverage and quality of care that is appropriate for a range of providers in primary and community settings in humanitarian contexts [18,19]. The app is an innovative and scalable approach that showed potential to improve adherence to guidelines and quality of care during development in Lebanon [18,19]. This study tested a modified version of the Sana mHealth app at the NCD clinic based in the Hagadera refugee camp hospital, which is supported by the International Rescue Committee (IRC). Established in 1992, the Hagadera camp is 1 of 3 camps in the Dadaab refugee camp complex, which collectively hosts more than 215,000 Somali refugees [20]. NCDs account for 24% and 27% of all deaths in Somalia and Kenya, respectively, and the camp context represented a stable location with high levels of need where the mHealth app could be tested [21].

This work aimed to evaluate a specialized mHealth app consisting of an abbreviated medical record for patients with hypertension or diabetes, adapted for a Kenyan refugee camp setting.

## Methods

### Study Design

A quantitative and qualitative prospective evaluation with an 11-month follow-up period (May 1, 2021, through March 31, 2022) was used to evaluate a version of the Sana.NCD mHealth app, adapted for the Hagadera refugee camp in Kenya.

### Intervention Description and Study Implementation

The Sana.NCD mHealth app, as originally designed and tested in Lebanon, included a patient-controlled health record in addition to serving as a diabetes and hypertension EMR and a decision support tool for providers. In this pilot study, the app was adapted for the localized context based on input from clinicians who would be using the app and IRC health program staff. The mHealth app evaluated in this study was an abbreviated medical record and clinical protocol for hypertension and diabetes; a patient-controlled health record was not relevant in this context because patients returned to the same location for care. For this pilot study, the app was integrated with the CommCare platform (Dimagi, Inc) [22], which was already used by the IRC and advantageous for future scale-up. MIT Sana and Dimagi worked in partnership with the IRC to customize the app to the workflows and context of IRC-supported health facilities in Hagadera. Providers were trained by Dimagi on app use, which was followed by a 2-week pilot period wherein the IRC team continued to practice using the app prior to the formal data collection period. Technical support was provided to the health facility for the duration of the study implementation period. Images of the Sana.NCD app interface are provided in Multimedia Appendix 1.

The planned study involved a comparison of the patients and sites supported by the IRC through the Sana.NCD app (Hagadera refugee camp) to those without the Sana.NCD app (Kakuma refugee camp, which hosts predominantly South Sudanese refugees); however, an unanticipated change to recordkeeping at the comparison facilities necessitated a design change. The IRC began the rollout of an outpatient register (an individual EMR) for all patients in the Kakuma refugee camp that facilitated reporting to the Ministry of Health of Kenya and the Integrated Refugee Health Information System [23] on May 1, 2021. Given the adoption of the EMR at Kakuma at a similar time as the use of the Sana.NCD app at Hagadera, the evaluation was revised to leverage the opportunity to compare the use of a specialized app for NCD management to the use of a more general EMR.

The study’s quantitative component consisted of secondary descriptive analysis of deidentified data collected in the Sana.NCD app for patients with hypertension and diabetes who received outpatient care at the Hagadera refugee camp hospital, in addition to EMR data provided by the IRC for patients visiting the IRC Kakuma hospital’s surgical and medical outpatient clinic departments (where patients with NCD are seen). Data from a total of 1539 patients in Hagadera using the Sana.NCD mHealth app and 68 patients (identifiable as having diabetes or hypertension) in the Kakuma comparison facilities with the EMR were analyzed. As Kakuma’s EMR system was newly implemented during the study period, reporting on several key indicators critical to diagnosis were frequently not captured or recorded in open-text “notes” fields, hindering the ability to quickly identify patients with specific health conditions. This limited the sample size from Kakuma and precluded more complex analyses comparing health outcomes between the intervention and comparison facilities, which was a planned study outcome.

The study’s qualitative component consisted of key informant interviews with IRC health facility staff that focused on their professional opinions of the app. All 6 health facility staff involved in providing NCD care who had experience using the Sana.NCD app participated in the key informant interviews. Interviews were conducted remotely by study team members who had no previous relationships with the key informants. Notes were taken during each interview, but no recordings were taken. Because the interviewers had no relationship with the key informants prior to the interview and they were not associated with the health facility where the participants were employed, perceived influence or conflict of interest is believed to be minimal. A structured facilitation guide was used; however, interviews were iterative, allowing for deeper probing of topics where needed. Interview content focused on the challenges faced in the use and adoption of the app, changes in NCD care and service provision resulting from the use of the app, changes in patient data collection and management and if this informed changes to patient care, and recommendations for future modifications to the app.

### Data Analysis

Quantitative data were analyzed using Stata 15 (StataCorp LLC). Descriptive statistics (means and proportions) were used to characterize the adoption of the app, compare reporting quality between the Hagadera (NCD app) and Kakuma (EMR) health facilities, and compare the completeness of reporting in Hagadera NCD app records between the first and last study quarters. Change from the first to last quarter of the study was also analyzed for key biometrics, namely BMI classification, elevated blood pressure, and elevated blood sugar. BMI was categorized as normal (<25 kg/m^2^), overweight (25-29.9 kg/m^2^), or obese (≥30 kg/m^2^); blood pressure was classified as normal (systolic<140 mm Hg and diastolic<90 mm Hg) or elevated (systolic>140 mm Hg or diastolic>90 mm Hg); and blood sugar was similarly classified as having achieved target levels (HbA_1c_<7.0% or random blood sugar<11.1 mmol/L, with preference given to HbA_1c_ where available) or elevated [24]. Linear probability models were used to estimate differences in biometric changes from the first to last study quarter between male and female patients and across patient age groups (<60 y, 60-69 y, and 70 y or older), with main terms for sex or age and study quarter and the interaction between sex or age and study quarter.

Qualitative key informant interview data (NCD app, Hagadera refugee camp only) were analyzed using thematic analysis, focusing on various process aspects of mHealth app use or adoption and user perceptions of changes in health service provision that may have resulted from the adoption of the app. Qualitative findings were used to contextualize trends in quantitative data, obtain a more nuanced understanding of the app’s utility, and provide recommendations on possible adaptations for the mHealth app and deployment strategies for scale-up in other contexts.

### Ethical Considerations

The Johns Hopkins School of Public Health Institutional Review Board reviewed this study and determined that it was not engaged in human research: only deidentified health program data were used for quantitative analysis, and interaction with human participants was limited to key informant interviews with health professionals and their experiences of using the technologies. Consent discussions for key informants occurred at the start of each interview, at which time key informants were read a consent statement and oral consent was obtained before proceeding with the interview.

## Results

### Facilities Comparison

The NCD clinic where the NCD app was implemented is situated at the outpatient department of the main Hagadera refugee camp hospital. The clinic operates from 8 AM to 4 PM, 6 days a week, with most patients seen in the morning and a few walk-ins in the afternoon. All new or suspected NCD cases are referred from other service points or by community health workers. All bookings are done at the clinic with a typical follow-up time of 1 month. The clinic is generally staffed by 1 clinician with additional support when needed. The comparison site covered by the EMR consists of 5 health facilities (including 1 camp hospital) in the Kakuma refugee camp. These clinics operate Monday through Friday and, as with the Hagadera NCD clinic, are staffed by 1 clinician per facility. Bookings are done independently in each facility on a daily basis, with specific “clinic days” for each condition. NCD services in Kakuma are decentralized and integrated with primary health care to enable patients to have easy access to NCD care in the facility closest to their residence. Care for chronic conditions is provided free of charge at both Hagadera and Kakuma health facilities.

During the 11-month study period, 1539 patients with diabetes or hypertension were seen on an outpatient basis in Hagadera and reported in the NCD app. Patients were predominantly female (n*=*928, 60.3%) and had an average age of 53 (range 6-100) years. The majority (n=898, 58.3%) were seen for hypertension, with diabetes and combined diabetes and hypertension diagnoses accounting for 23.2% (n=357) and 18.5% (n=284) of patients, respectively (Table 1). The identification of patients with hypertension or diabetes was a challenge in the EMR due to the absence of consistently used fields for reporting the diagnosis. Consequently, most patients with these conditions were not captured by this study; the 68 patients that are included were identified based on information provided in text fields. Demographic characteristics of the included Kakuma care seekers were similar in that the population was predominantly female (n=52, 77%) and had a similar age distribution (mean 53; range 15-95 y). A notable difference in diagnosis was observed, with 96% (n=65) of Kakuma patients having hypertension and small numbers of patients having only diabetes (n=2, 3%) or both diabetes and hypertension (n=1, 2%). There is, however, a substantial bias in this distribution that becomes evident when caseload is considered, which is difficult to quantify. Hagadera reported 18,801 visits to the NCD clinic during the study period, of which 42.1% (n=7918) were reported in the NCD app. In Kakuma, a total of 350,776 visits to the medical and surgical outpatient departments were reported for a broader range of conditions; of these consultations, 9385 (2.7%) were for NCDs, including 4264 (1.2%) for hypertension and 2415 (0.7%) for diabetes, suggesting that the EMR did not aptly characterize patient diagnoses.

**Table 1.** Demographics and diagnosed condition(s) of patients with hypertension and diabetes.

Characteristic		NCD^a^ app in the Hagadera camp (n=1539)		EMR^b^ in the Kakuma camp (n=68)	
Age (years), mean (SD)^c^		52.6 (14.6)		52.6 (14.1)	
**Sex, n (%)**					
	Male	610 (39.7)		16 (23.5)	
	Female	928 (60.3)		52 (76.5)	
**Diagnosed condition(s) of interest, n (%)**					
	Hypertension only	898 (58.3)		65 (95.6)	
	Diabetes only	357 (23.2)		2 (2.9)	
	Hypertension and diabetes	284 (18.5)		1 (1.5)	

a NCD: noncommunicable disease.

b EMR: electronic medical record.

c NCD app: n=1501; EMR: n=68.

Reporting frequency for key information of interest (eg, relevant biometric data, comorbidities, and risk factors) was assessed as a measure of the quality of care and is summarized in Table 2. Patient age, sex, prescribed medicines, random blood sugar (among patients with diabetes), and smoking status were consistently reported in both the NCD app and the EMR. In contrast, reporting of comorbidities, complications, HbA_1c_ (the gold-standard metric of longer-term glycemic control), and diet were rarely reported in either platform. Indicators frequently reported in the NCD app but not the EMR included the number of health facility visits, BMI, blood pressure, smoking history, physical activity, and next follow-up visit. As previously noted, figures for the Kakuma EMR should be interpreted with caution given that a substantial number of patients with NCD were likely not captured by this study.

**Table 2.** Reporting quality: the proportion of records with key data reported (full study period).

Record			NCD^a^ app in the Hagadera camp (n=7918), n (%)		EMR^b^ in the Kakuma camp (n=68), n (%)	
**Patient demographics**						
	Age		7801 (98.5)		68 (100)	
	Sex		7908 (99.9)		68 (100)	
**Clinical history and presentation**						
	Comorbidities		219 (2.8)		0 (0)	
	Complications^c^		248 (3.1)		0 (0)	
	Number of visits to facility		7839 (99)		0 (0)	
**Any prescribed medicine(s**)						
	Among all patients		7834 (98.9)		68 (100)	
	Among patients with hypertension only^d^		6105 (100)		66 (100)	
	Among patients with diabetes only^e^		3432 (100)		3 (100)	
**Biometrics**						
	BMI		7868 (99.4)		0 (0)	
	Height		7868 (99.4)		0 (0)	
	Weight		7918 (100)		5 (7.4)	
	**Blood pressure**					
		Both systolic and diastolic reported	7918 (100)		51 (75)	
		Systolic only	7918 (100)		0 (0)	
		Diastolic only	7918 (100)		0 (0)	
		Neither systolic nor diastolic reported	0 (0)		17 (25)	
	**Blood sugar**					
		Random blood sugar (all patients)	3505 (44.3)		6 (8.8)	
		Random blood sugar (patients with diabetes)^e^	3368 (98.1)		3 (100)	
		Hemoglobin A_1c_ (patients with diabetes)^e^	10 (0.3)		0 (0)	
**Lifestyle risk factor**						
	**Smoking**					
		Current smoking status	7839 (99)		68 (100)	
		History of tobacco intake	7839 (99)		0 (0)	
	Physical activity or sedentary lifestyle		7843 (99.1)		0 (0)	
	**Diet**					
		Salt intake	522 (6.6)		0 (0)	
		Carbohydrate intake	81 (1)		0 (0)	
		Protein intake	10 (0.1)		0 (0)	
		Fat intake	2 (0)		0 (0)	
Follow-up: next care visit date			7884 (99.6)		10 (14.7)	

a NCD: noncommunicable disease.

b EMR: electronic medical record.

c Complications include amputation, blindness, stroke, end-stage renal disease requiring dialysis, and other.

d NCD app: n=6105; EMR: n=66.

e NCD app: n=3432; EMR: n=3.

### Reporting Quality Changes Over Time

The completeness of reporting was used as a metric of the quality of care, where having longitudinal information can be important for decision-making in chronic conditions care. Due to the limited Kakuma EMR sample size (first study quarter n=4; last study quarter n=28), reporting quality over time was evaluated only for the NCD app. A review of the paper record system that was in place in Hagadera prior to the adoption of the NCD app found numerous gaps in the information available in patient charts as well as inconsistencies in reporting across visits. Given that one of the primary aims of using the app was improved reporting, changes in reporting were tracked over time; reporting rates for the first and last quarters of the NCD app implementation period are presented in Table 3. Completeness in reporting was consistently high across the implementation period, with little meaningful change over time. Nearly all fields were reported for ≥98% of patients each quarter. The exceptions were the reporting of complications (<4%), comorbidities (<3%), and diet (<6%), which were due to the data entry structure. Complications and comorbidities were entered through a multicheckbox question, with checkboxes for each complication or comorbidity observed but no checkbox for “none observed.” Diet was captured using an open-text question in a patient’s medical history, but according to information shared during qualitative interviews with the providers, most clinicians left the field blank because it did not include specific guidance on what to include as part of “diet and salt intake.”

**Table 3.** Completeness of reporting in the Hagadera noncommunicable disease app records in first and last study quarters.

Record			First quarter (May to July 2021; n=2380), n (%)		Last quarter (January to March 2022; n=1645), n (%)	
**Patient demographics**						
	Age		2367 (99.5)		1604 (97.5)	
	Sex		2380 (100)		1643 (99.9)	
**Clinical history and presentation**						
	Comorbidities		63 (2.6)		56 (3.4)	
	Complications^a^		60 (2.5)		54 (3.3)	
	Number of visits to facility		2349 (98.7)		1633 (99.3)	
**Any prescribed medicine(s**)						
	Among all patients		2349 (98.7)		1630 (99.1)	
	Among patients with hypertension only^b^		1860 (100)		1242 (100)	
	Among patients with diabetes only^c^		1052 (100)		692 (100)	
**Biometrics**						
	BMI		2367 (99.5)		1623 (98.7)	
	Height		2367 (99.5)		1623 (98.7)	
	Weight		2380 (100)		1645 (100)	
	**Blood pressure**					
		Systolic	2380 (100)		1645 (100)	
		Diastolic	2380 (100)		1645 (100)	
	**Blood sugar (among patients with diabetes)**					
		Random blood sugar^c^	1027 (97.6)		679 (98.1)	
		Hemoglobin A_1c_^c^	0 (0)		2 (0.3)	
**Lifestyle risk factor**						
	**Smoking**					
		Current smoking status	2349 (98.7)		1633 (99.3)	
		History of tobacco intake	2349 (98.7)		1633 (99.3)	
	Physical activity or sedentary lifestyle		2352 (98.8)		1633 (99.3)	
	**Diet**					
		Salt intake	130 (5.5)		104 (6.3)	
		Carbohydrate intake	26 (1.1)		18 (1.1)	
		Protein intake	3 (0.1)		2 (0.1)	
		Fat intake	0 (0)		0 (0)	
Follow-up: next care visit date			2365 (99.4)		1639 (99.6)	

a Complications include amputation, blindness, stroke, end-stage renal disease, and other.

b First quarter: n=1860; last quarter: n=1242.

c First quarter: n=1052; last quarter: n=692.

### Hypertension and Diabetes Management

Given the ultimate goal of improving health outcomes, several key biometric indicators were analyzed to examine change from the first to last quarter of NCD app implementation (Table 4). Differences in these indicators and their change over time between male and female patients and across patient age groups are also provided in Multimedia Appendix 2. Mean BMI was nearly identical in the first and last study quarters. Although the proportion of patients classified as overweight or obese decreased by 3.4% (95% CI −7.3% to 0.6%; *P*=.09), the change was not statistically significant. Approximately half of patients had elevated blood pressure in the first (599/1094, 54.8%) and last (720/1395, 51.6%) study quarters, with an overall decrease of 3.1% (95% CI −7.1% to 0.8%; *P*=.12). Elevated glucose or blood sugar levels were reported for 62.8% (302/481) of patients overall in the first study quarter and 62.1% (374/602) in the last quarter, equating to a 0.7% (95% CI −6.5% to 5.1%; *P*=.82) decrease during the study period.

**Table 4.** BMI, blood pressure, and blood sugar levels among patients with hypertension or diabetes in the Hagadera camp.

		First quarter, value (95% CI)	Last quarter, value (95% CI)	Change over time	
				Value (95% CI)	*P* value
**BMI^a^^,^^b^**					
	Mean BMI (kg/m^2^)	25.2 (24.9 to 25.4)	25.1 (24.9 to 25.3)	N/A^c^	
	Normal (%)	54.5 (51.5 to 57.5)	57.9 (55.3 to 60.5)	N/A	
	Overweight (%)	34.4 (31.6 to 37.3)	31.1 (28.6 to 33.6)	N/A	
	Obese (%)	11.0 (9.2 to 12.9)	11.0 (9.3 to 12.7)	N/A	
	Overweight or obese (%)	N/A	N/A	−3.4 (−7.3 to 0.6)	.09
**Blood pressure^d^^,^^e^**					
	Normal (%)	45.2 (42.3 to 48.2)	48.4 (45.8 to 51.0)	N/A	
	Elevated (%)	54.8 (51.8 to 57.7)	51.6 (49.0 to 54.2)	−3.1 (−7.1 to 0.8)	.12
**Blood sugar^f^^,^^g^**					
	Target achieved (%)	37.2 (32.9 to 41.5)	37.9 (34.0 to 41.8)	N/A	
	Elevated (%)	62.8 (58.5 to 67.1)	62.1 (58.2 to 66.0)	−0.7 (−6.5 to 5.1)	.82

a Defined as <25 kg/m2=normal, 25-29.9 kg/m2=overweight, and ≥30 kg/m2=obese.

b First quarter: n=1086; last quarter: n=1373.

c N/A: not applicable.

d Normal blood pressure defined as systolic<140 mm Hg and diastolic<90 mm Hg; elevated blood pressure defined as systolic>140 mm Hg or diastolic>90 mm Hg.

e First quarter: n=1094; last quarter: n=1395.

f Among patients with diabetes and based on results from either random blood sugar (RBS) or hemoglobin A_1c_ (HbA_1c_; target blood sugar level defined as HbA_1c_<7.0% or RBS<11.1 mmol/L), with preference given to HbA_1c_ results (when available) or most recent RBS test in cases of multiple measures.

g First quarter: n=481; last quarter: n=602.

### Provider Perceptions

Overall, providers were satisfied with the app and indicated it standardized patient information and made consultations easier. There was consensus that access to information from previous consultations was easier when using the app and that this was beneficial for NCD control and follow-up. Prior to the implementation of the app, patient data were collected using a paper medical record, which was stored in file cabinets. Providers mentioned that these records frequently got lost and that the use of the app eliminated data loss and allowed for information to be stored securely. As one provider mentioned, “Most important aspect is the traceability of the data and the way the data is saved and stored. There is no problem if the patient loses their patient’s card or number.”

Although all providers were supportive of continued use of the app and felt that it was beneficial for multiple aspects of patient care, several challenges were observed. Duplication was a concern, where patients with NCD often have multiple diagnoses. For patients with multiple diagnoses, the app format required their information to be entered twice, which increased time requirements for providers. Another concern were field limits restricting minimum and maximum values for selected variables. These were intended to reduce data entry errors and improve data quality but proved problematic in the case of patients with outlying values. One provider shared the following: “When entering patients’ data, for example diabetic patients, with a weight of less than 50 kgs you can’t input that info…also, blood pressure has a cap, some patients with uncontrolled hypertension have high blood pressure which can’t be entered.” Another provider observation was the need for an updated monthly medication list within the app because medication availability and stock-outs can change monthly.

## Discussion

### Principal Findings

Thorough, consistent, and accessible reporting of patient data is essential for monitoring disease control and informing treatment decisions for patients with chronic NCD such as hypertension and diabetes. As the global NCD burden continues to rise and both the number of humanitarian crises and the scale of displacement increase, innovative solutions are needed to ensure access to and quality of care for NCDs, given the limitations and challenges in health service delivery in humanitarian contexts [4]. Although this study did not explicitly seek to assess the impact of the NCD app on clinical measures, it nevertheless provided important evidence of the successful implementation of an mHealth tool in the context of a refugee camp NCD clinic. Our findings demonstrate consistently higher reporting quality for patients with hypertension and diabetes in a specialized app for NCD management than a more general EMR. Additionally, positive provider perceptions further highlight several important facilitators of and barriers to effective uptake of an mHealth app such as the one evaluated.

EMR systems have meaningful potential to decrease care fragmentation and improve the continuity of care [25]. In conditions such as diabetes and hypertension, disease management, responsive treatment decisions, and the prevention of complications require continuous tracking of clinical measures such as blood sugar, blood pressure, and BMI, as well as timely access to historical patient data [26,27]. The NCD app tested at Hagadera provided valuable evidence of improved patient data tracking, follow-up, and continuity of care. Providers at Hagadera also indicated that by improving access to patients’ data, the tool reduced distraction during consultations; this led to improved patient-provider interactions, which is another critical aspect of the effective management of chronic NCDs [7,26].

When implementing a digital tool such as the app in Hagadera, integration into the existing health care service environment is important to consider, as uptake requires providers to adopt and regularly use the tool and for patients to accept its use [6,27,28]. The time required to learn how to use an mHealth tool and the ease of use during appointments are decisive factors for the adoption of new technologies due to the overburden that health care providers often confront in their daily work [29,30]. In the case of the Hagadera experience, the app originally developed for use in Lebanon was substantially modified to meet the localized context in Kenya. Health providers’ feedback on the app’s utility and the consistently high reporting of patient data within the NCD app demonstrate the benefit of collaborating with users throughout the app’s design and implementation, to create a tool that is not only acceptable but also meets users’ specific needs and is feasible to implement in day-to-day clinical practice.

Successful deployment of an mHealth app, especially one specified for particular conditions, also requires integration into existing digital ecosystems, which is critical for both efficiency and long-term sustainability. Carefully designed systems integration is essential for uptake and reporting consistency in facilities with digital technologies such as EMRs or other health information systems previously in place [8]. When implemented correctly, this can reduce data redundancy and duplicated data entry, as well as improve reporting capabilities. Although this may be perceived as adding to providers’ workload, feedback from users of the NCD app in Hagadera regarding the app’s utility and usability suggest that by actively involving end users in the design of the app and using a platform already known to the IRC, the work required to integrate with existing systems was not an influential barrier to the app’s use.

One advantage of most mHealth apps is that changes and improvements can be made incrementally. Although a common theme in the qualitative interviews was the Hagadera NCD app’s ease of use, several relatively straightforward changes (eg, restrictions on permissible values for entering clinical measures, such as weight, height, and blood pressure, and regularly updating listed medication options to reflect availability) were identified and put in place at the end of the pilot period to improve patient information input and user experience. The ease with which the app can be modified also facilitates interoperability with other tools, applications, and reporting requirements, which can in turn facilitate its utility and benefits [7,27]. Being able to adapt the app to meet reporting requirements more efficiently is particularly valuable in humanitarian contexts, where those providing services often have to report various information to a range of bodies (eg, government, donor, and intraorganizational). With the increasing use of electronic health information systems, the ability to design and subsequently easily adapt mHealth tools to align with existing or new tools and applications is invaluable for ensuring consistency in reporting and the continuity of quality health care, especially in settings with multiple service providers and actors providing or supporting health services, such as in humanitarian crises.

Finally, the ethical aspects of the use of mHealth tools and other digital interventions must be considered due to the sensitive and personally identifiable information that is managed within these systems. Data confidentiality is a critical concern, and in the case of Hagadera, the app appeared to improve secure data collection and storage practices. Data storage within remote or local servers with password-protected access was perceived as an improvement over paper records.

### Limitations

Despite the challenges to conducting rigorous research in humanitarian settings, evaluations such as this are nevertheless valuable in contributing to the limited evidence available to inform health services in such contexts. The change in study design necessitated by the unanticipated implementation of an EMR system at the Kakuma health facilities limited the planned comparison and subsequent analyses. The ideal comparison would have been a facility with a paper-based system; however, given increasing use of electronic systems for health information, comparing 2 electronic systems is nevertheless valuable. This evaluation was critically limited by the small number of patient records available for the comparison facilities at Kakuma. This hindered the ability to compare reporting quality over time in both sites and to examine health outcomes among patients with data in the Kakuma EMR. Another limitation was the length of the follow-up period with respect to examining changes in blood pressure and blood sugar control, where longer time periods and more robust metrics (eg, repeat blood pressure measurements, HbA_1c_, or longitudinal fasting blood sugar readings for blood sugar) are necessary to analyze disease control. Finally, the evaluation approach used quantitative patient data and qualitative provider data but did not capture patients’ perspectives on the app’s use and if or how it affected interactions at health facilities or the quality of care.

### Conclusions

Due demographic transitions and the increasing global NCD burden, more attention to the management of these conditions in complex settings with limited resources is critical. Given the growing burden of NCDs, strategies to increase the quality, continuity, and effectiveness of care that are relevant to primary care settings are urgently needed. In this study of EMR adoption in Kenyan refugee settings, a specialized record for NCDs outperformed a more general record intended for use in all patients in terms of reporting completeness. The demonstrated feasibility of high-quality, standardized routine data collection for key health parameters is a critical step toward improving the quality and continuity of care, but it was insufficient to improve blood pressure and blood sugar during the study period. Improving NCD care and outcomes in humanitarian and low-income settings will be a long-term and multifaceted undertaking. Enhanced recordkeeping is feasible, is valued by providers, and is an important first step to improving the quality of care. This CommCare-based NCD app can easily be rolled out in similar humanitarian settings with minimal adaptation; however, the adaptation of technologies to the local context and use case is critical for uptake and ensuring that workflows and time burden do not outweigh the benefits of EMRs.

## Supplementary material

10.2196/43878 Multimedia Appendix 1 Images of the Sana.NCD mobile health app interface.

10.2196/43878 Multimedia Appendix 2 BMI, blood pressure, and blood sugar levels among patients with hypertension or diabetes in the Hagadera camp by patient sex and age group.

## Abbreviations

EMR: electronic medical record
HbA_1c_: hemoglobin A_1c_
IRC: International Rescue Committee
mHealth: mobile health
MIT: Massachusetts Institute of Technology
NCD: noncommunicable disease
UNRWA: United Nations Relief and Works Agency for Palestine Refugees in the Near East
