# Supplementary material for: Adoption of Electronic Medical Records for Chronic Disease Care in Kenyan Refugee Camps: Quantitative and Qualitative Prospective Evaluation
Source: JMIR Mhealth Uhealth. 2023 Oct 5;11:e43878. doi: 10.2196/43878 (PMC10578110; doi:10.2196/43878)
Supplement: Multimedia Appendix 1 [file mhealth-v11-e43878-s001.pdf]

Multimedia Appendix 2: Images of Sana.NCD mHealth Application Interface

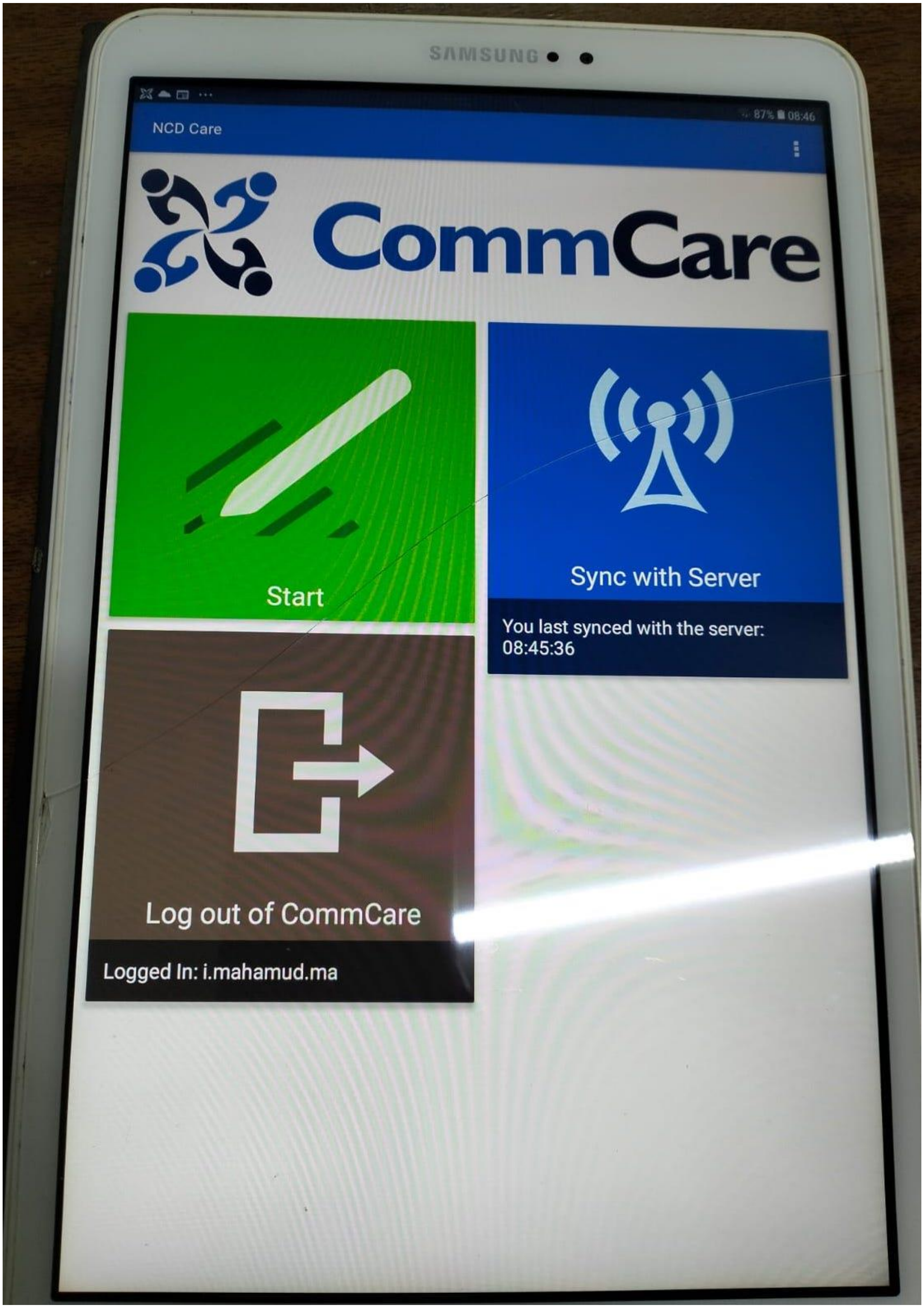

Image 1: Main screen upon opening Sana.NCD mHealth application

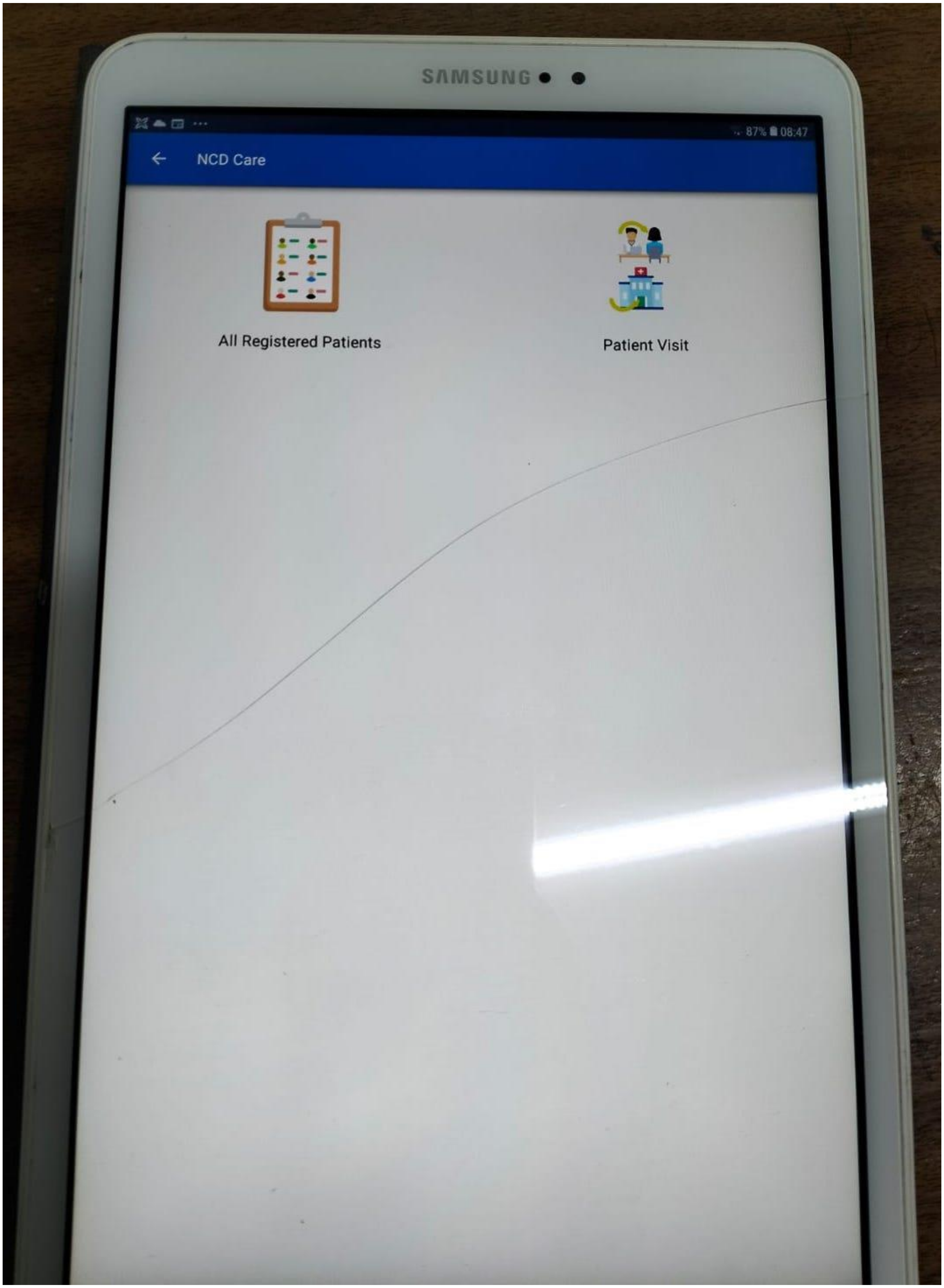

Image 2: Menu directing user to access patient list and data entry

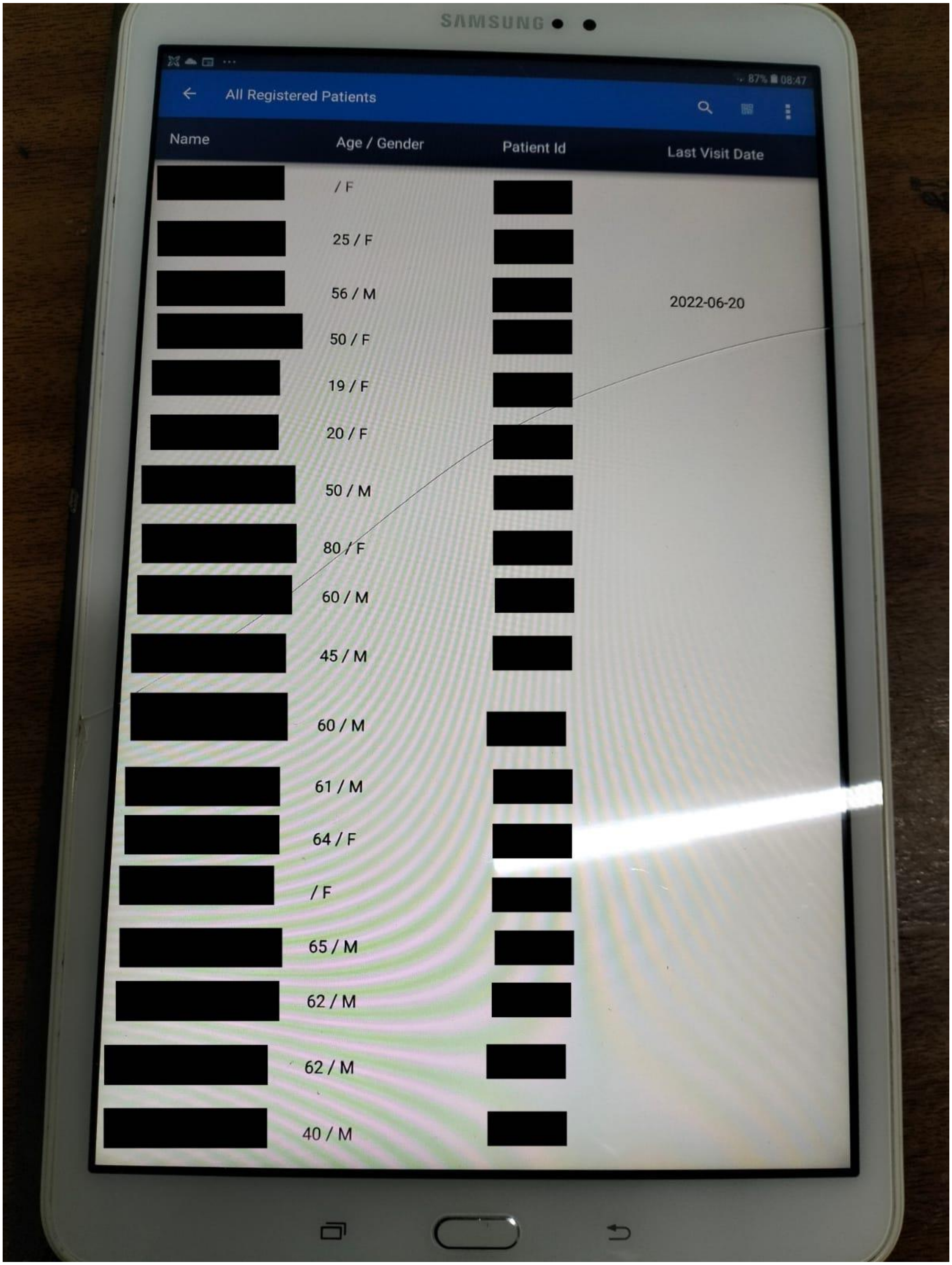

Image 3: Main list of registered patients and key information (i.e., patients' name, age/sex, identification number, and date of last visit)

[personally identifiable information has been censored for patient privacy]

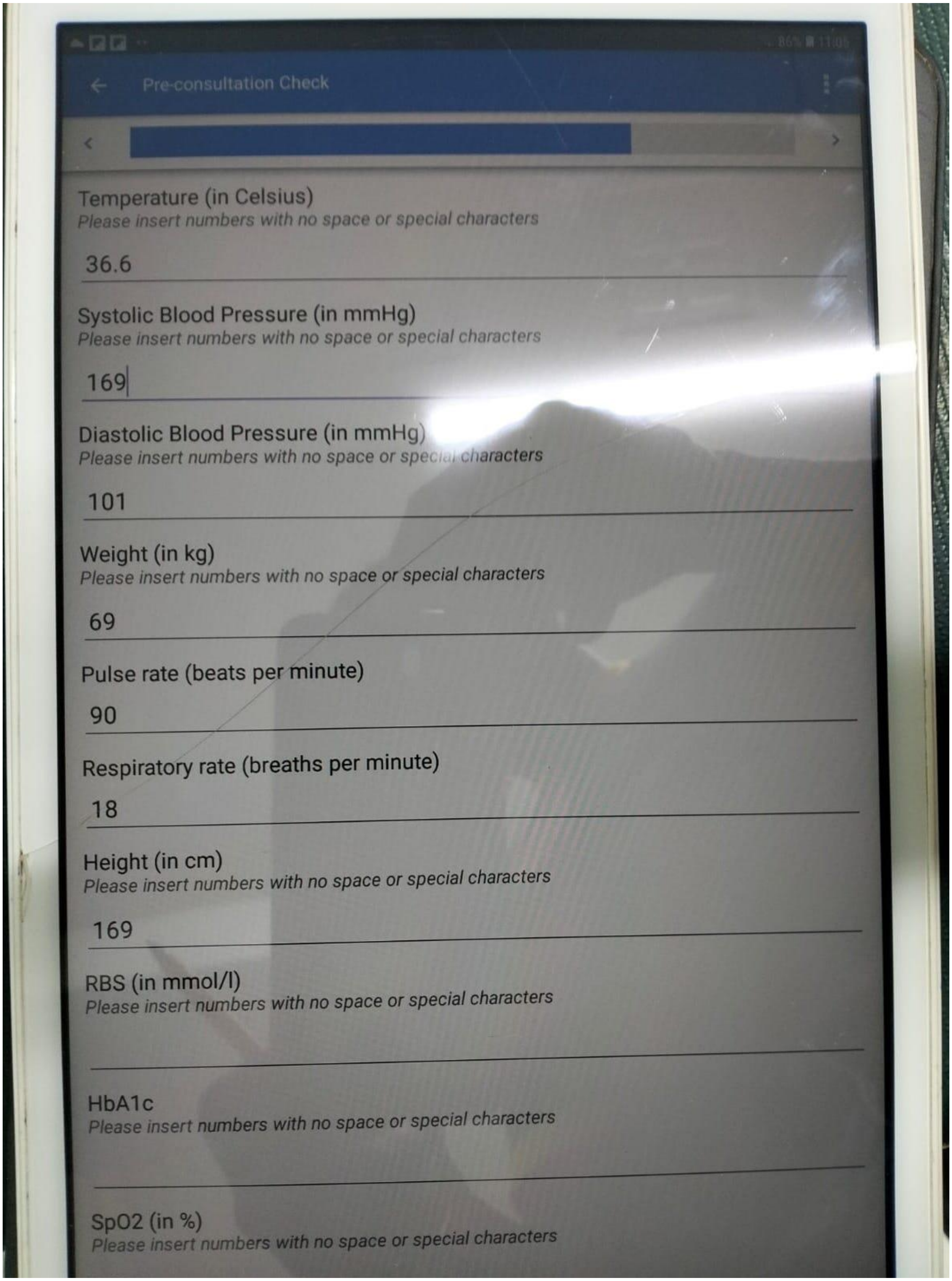

Image 4: Pre-consultation check screen where clinic staff enter vitals and relevant test results

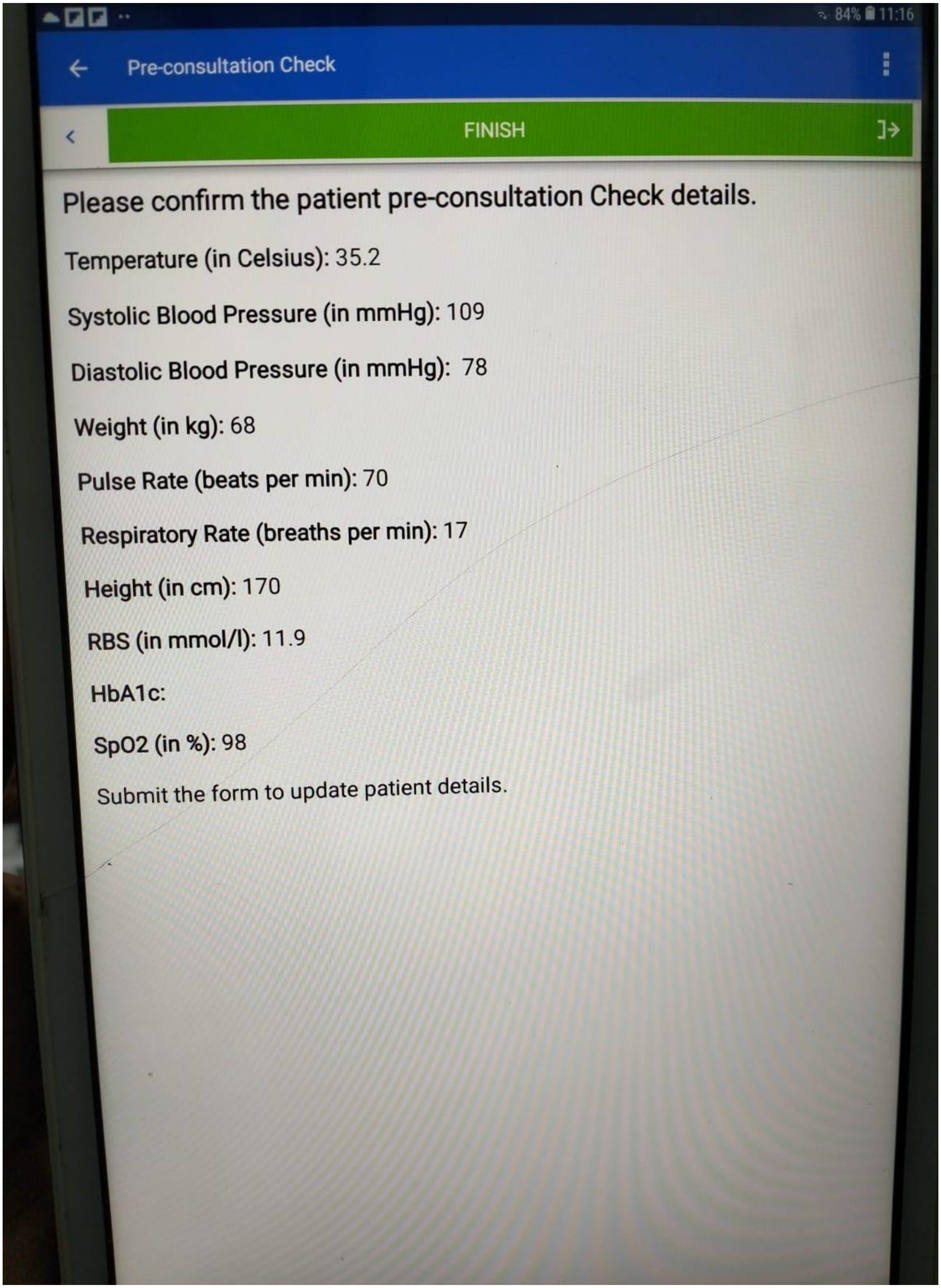

Image 5: Pre-consultation check confirmation screen where clinic staff are prompted to review and confirm data entered in the pre-consultation check
