# Supplementary material for: Adoption of Electronic Medical Records for Chronic Disease Care in Kenyan Refugee Camps: Quantitative and Qualitative Prospective Evaluation
Source: JMIR Mhealth Uhealth. 2023 Oct 5;11:e43878. doi: 10.2196/43878 (PMC10578110; doi:10.2196/43878)
Supplement: Multimedia Appendix 2 [file mhealth-v11-e43878-s002.pdf]

Body Mass Index, Blood Pressure, and Blood Sugar Levels Among Patients with Hypertension and/or Diabetes in Hagadera Camp by Patient Sex and Age Group

	By Age Group			*P*-value[a]	By Sex		*P*-value[a]
	<60 years	60-69 years	70+ years		Males	Females	
**First Quarter**							
**Body Mass Index** [b]	*n=719*	*n=218*	*n=140*		*n=415*	*n=671*	
Mean BMI	25.4 (25.1-25.7)	24.9 (24.4-25.5)	24.6 (24.0-25.3)	.08	24.4 (24.0-24.7)	25.7 (25.4-26.0)	<.001
Normal	50.5% (46.8-54.2%)	59.6% (53.1-66.2%)	65.7% (57.8-73.7%)	.001	63.1% (58.5-67.8%)	49.2% (45.4-53.0%)	<.001
Overweight	36.9% (33.3-40.4%)	33.9% (27.6-40.3%)	24.3% (17.1-31.5%)		31.1% (26.6-35.6%)	36.5% (32.9-40.2%)	
Obese	12.7% (10.2-15.1%)	6.4% (3.1-9.7%)	10.0% (5.0-15.0%)		5.8% (3.5-8.0%)	14.3% (11.7-17.0%)	
**Blood Pressure** [c]	n=723	n=219	n=143		*n=417*	*n=677*	
Normal	48.3% (44.6-51.9%)	40.2% (33.6-46.7%)	37.1% (29.1-45.1%)	.01	45.6% (40.8-50.4%)	45.1% (41.3-48.8%)	.87
Elevated	51.7% (48.1-55.4%)	59.8% (53.3-66.4%)	62.9% (54.9-70.9%)		54.4% (49.6-59.2%)	54.9% (51.2-58.7%)	
**Blood Sugar** [d]	n=350	n=80	n=47		*n=195*	*n=286*	
Target achieved	35.1% (30.1-40.2%)	42.5% (31.4-53.6%)	46.8% (32.0-61.6%)	.18	43.1% (36.1-50.1%)	33.2% (27.7-38.7%)	.03
Elevated	64.9% (59.8-69.9%)	57.5% (46.4-68.6%)	53.2% (38.4-68.0%)		56.9% (49.9-63.9%)	66.8% (61.3-72.3%)	
**Last Quarter**							
**Body Mass Index** [b]	n=882	n=277	n=185		n=548	n=823	
Mean BMI	25.2 (24.9-25.5)	25.4 (24.8-25.9)	24.4 (23.9-24.8)	.03	24 (23.8-24.3)	25.8 (25.5-26.2)	<.001
Normal	55.7% (52.4-59.0%)	57.4% (51.5-63.3%)	69.2% (62.5-75.9%)	.008	67.0% (63.0-70.9%)	51.8% (48.3-55.2%)	<.001
Overweight	32.2% (29.1-35.3%)	33.9% (28.3-39.5%)	22.7% (16.6-28.8%)		28.1% (24.3-31.9%)	33.2% (29.9-36.4%)	
Obese	12.1% (10.0-14.3%)	8.7% (5.3-12.0%)	8.1% (4.1-12.1%)		4.9% (3.1-6.7%)	15.1% (12.6-17.5%)	
**Blood Pressure** [c]	n=892	n=286	n=188		n=559	n=834	
Normal	52.6% (49.3-55.9%)	39.5% (33.8-45.2%)	39.4% (32.3-46.4%)	<.001	46.5% (42.4-50.7%)	49.5% (46.1-52.9%)	.27
Elevated	47.4% (44.1-50.7%)	60.5% (54.8-66.2%)	60.6% (53.6-67.7%)		53.5% (49.3-57.6%)	50.5% (47.1-53.9%)	
**Blood Sugar** [d]	n=446	n=101	n=39		n=252	n=350	
Target achieved	36.5% (32.1-41.0%)	40.6% (30.9-50.3%)	41.0% (24.9-57.2%)	.67	41.3% (35.1-47.4%)	35.4% (30.4-40.5%)	.15
Elevated	63.5% (59.0-67.9%)	59.4% (49.7-69.1%)	59.0% (42.8-75.1%)		58.7% (52.6-64.9%)	64.6% (59.5-69.6%)	
**Change Over Time** [c]	**<60 years**	**60-69 years**	**70+ years**		**Males**	**Females**	
Overweight or obese	−5.2% (−10.1- −0.3%)	2.2% (−6.5-11.0%)	−3.5% (−13.8-6.8%)		−3.8% (−9.9-2.2%)	-2.6% (−7.7-2.5%)	
Elevated blood pressure	−4.3% (−9.2-0.6%)	0.7% (−7.9-9.3%)	−2.3% (−12.9-8.3%)		−0.9% (−7.3-5.4%)	-4.5% (−9.5-0.6%)	
Elevated blood sugar	−1.4% (−8.1-5.3%)	1.9% (−12.6-16.4%)	5.8% (−15.2-26.8%)		1.8% (−7.4-11.0%)	-2.2% (−9.6-5.2%)	

[a] Comparison of differences by age/sex within the study quarter
[b] Defined as <25kg/m2=normal, 25-29.9kg/m2 = overweight, ≥30kg/m2 = obese
[c] Normal blood pressure defined as systolic <140 and diastolic <90; elevated blood pressure defined as systolic >140 and/or diastolic >90
[d] Among patients with diabetes and based on results from either RBS or HbA1c (target blood sugar level defined as HbA1C<7.0% or RBS<11.1 mmol/L) with preference given to HbA1c results (when available) or most recent RBS test in cases of multiple measures

BMI was higher for female patients than males in both quarters, and while the proportion of patients classified as overweight or obese decreased in both men (−3.8%, CI: −9.9-2.2%) and women (−2.6%, CI: −7.7-2.5%), these changes were not statistically significant. Average BMI was similar across age groups in the first quarter but was significantly lower in the last quarter for patients 70+ years of age than in other age groups. The proportion of overweight/obese patients changed differentially based on patient age—patients less than 60 years old saw the only statistically significant change, decreasing by 5.2% (CI: −10.1- -0.3%), whereas patients 70 years and older saw a 3.5% (CI: −13.8-6.8%) decrease and those 60-69 years saw an increase of 2.2% (CI: −6.5-11.0%).

While similar proportions of male and female patients had elevated blood pressure in both quarters, elevated blood pressure decreased more among female patients (−4.5%, CI: −9.5-0.6%) than males (−0.9%,

CI: −7.3%-5.4%), though changes were not statistically significant. Elevated blood pressure was increasingly more common in with older age groups and those less than 60 years old saw the greatest decrease of 4.3% (CI: −9.2-0.6%), compared to a 2.3% (CI: −12.9-8.3%) decrease in those 70 or older and a 0.7% (CI: −7.9-9.3%) increase in patients 60-69 years, though these changes were again not statistically significant.

Similar proportions of male and female patients had elevated blood sugar with a 1.8% (CI: −7.4-11.0%) increase among male patients during the study period but a 2.2% (CI: −9.6-5.2%) decrease among females, both of which were not statistically significant. The prevalence of elevated blood sugar was also similar across age groups with notable, though not statistically significant, changes over time—the proportion of those 70 years or older with elevated blood sugar increase 5.8% (CI: −15.2-26.8%), those 60-69 years increased 1.9% (CI: −12.6-16.4%), and patients less than 60 years decreased 1.4% (CI: −8.1-5.3%).
